# Annual Address before the Medical Society of Georgia

**Published:** 1858-05

**Authors:** 


### Annual Address before the Medical Society of Geo.

The Annual Address before the Medical Society of Georgia was delivered by Prof. Thos. S. Powell, of the Atlanta Medical College.

The subject of the address was, the "Obstacles in the way of the advancement of the Medical Profession," which were stated by the speaker, as mainly, *selfishness* and *indolence.*

These difficulties were strikingly set forth, and were clearly exhibited as greatly interfering with the harmony and progress of the profession.

Altogether, the address presented a high moral tone, and abounded in liberal and enlarged views, highly creditable to the speaker.

